# Epidemiological and Genomic Analysis of SARS-CoV-2 in 10 Patients From a Mid-Sized City Outside of Hubei, China in the Early Phase of the COVID-19 Outbreak

**DOI:** 10.3389/fpubh.2020.567621

**Published:** 2020-09-18

**Authors:** Jinkun Chen, Evann E. Hilt, Fan Li, Huan Wu, Zhuojing Jiang, Qinchao Zhang, Jiling Wang, Yifang Wang, Ziqin Li, Jialiang Tang, Shangxin Yang

**Affiliations:** ^1^Shaoxing Center for Disease Control and Prevention, Shaoxing, China; ^2^Department of Pathology and Laboratory Medicine, University of California, Los Angeles, Los Angeles, CA, United States; ^3^Three Coin Analytics, Inc., Pleasanton, CA, United States; ^4^IngeniGen XunMinKang Biotechnology Inc., Shaoxing, China; ^5^Zhejiang-California International Nanosystems Institute, Zhejiang University, Hangzhou, China

**Keywords:** 2019-nCoV, COVID-19, genotype, metagenomic sequencing, mutation rate, transmission, genomic epidemiology, SARS-CoV-2

## Abstract

A novel coronavirus known as severe acute respiratory syndrome coronavirus 2 (SARS-CoV-2) is the cause of the ongoing Coronavirus Disease 2019 (COVID-19) pandemic. In this study, we performed a comprehensive epidemiological and genomic analysis of SARS-CoV-2 genomes from 10 patients in Shaoxing (Zhejiang Province), a mid-sized city outside of the epicenter Hubei province, China, during the early stage of the outbreak (late January to early February, 2020). We obtained viral genomes with >99% coverage and a mean depth of 296X demonstrating that viral genomic analysis is feasible via metagenomics sequencing directly on nasopharyngeal samples with SARS-CoV-2 Real-time PCR C_t_ values <28. We found that a cluster of four patients with travel history to Hubei shared the exact same virus with patients from Wuhan, Taiwan, Belgium, and Australia, highlighting how quickly this virus spread to the globe. The virus from another cluster of two family members living together without travel history but with a sick contact of a confirmed case from another city outside of Hubei accumulated significantly more mutations (9 SNPs vs. average 4 SNPs), suggesting a complex and dynamic nature of this outbreak. Our findings add to the growing knowledge of the epidemiological and genomic characteristics of SARS-CoV-2 and offers a glimpse into the early phase of this viral infection outside of Hubei, China.

## Introduction

Coronaviruses (CoVs) are a large family of single-stranded RNA viruses that can be isolated from a variety of animals including camels, rats, birds, and bats (1). These coronaviruses can cause a range of disease states in animals including respiratory, enteric, hepatic, and neurological disease (2). Before late 2019, there were six known CoVs capable of infecting humans (Hu-CoVs). The first four Hu-CoVs that cause mild disease are HKU1, NL63, OC43, and 229E and are known to circulate in the human population (3). The other two Hu-CoVs, known as severe acute respiratory syndrome-CoV (SARS-CoV) and middle east respiratory syndrome-CoV (MERS-CoV), caused two previous epidemics in 2003 (4) and 2012 (5), respectively. Both SARS-CoV and MERS-CoV were the results of recent spillover events from animals. These two epidemics highlighted how easy it is for spillover events in CoVs to occur and cause outbreaks in humans.

In December 2019, another spillover event occurred and a seventh Hu-CoV appeared known as severe acute respiratory syndrome coronavirus 2 (SARS-CoV-2), previously named 2019-nCoV (6). SARS-CoV-2 has been spreading rapidly across the world since it was first reported in Wuhan, Hubei province, China (6, 7). The advances and accessibility of sequencing technologies have allowed researchers all over the world to quickly sequence the genome of SARS-CoV-2 (8, 9). Zhou et al. (9) showed that SARS-CoV-2 shared 79.6% sequence identity to SARS-CoV and 96% sequence identity to a bat CoV further supporting the theory of another spillover event.

Genomic analysis of SARS-CoV-2 genomes suggested that there were two major genotypes in the early phase of the outbreak, known as L type and S type, based on almost complete linkage between two SNPs (10). Tang et al. (10) proposed that the S type was more ancient while the L type evolved later and may be more aggressive in replication rates and spreads more quickly. Recent reclassifications of SARS-CoV-2 proposed the use of clade nomenclature and divided all viral genomes into 7 major clades including O, S, L, V, G, GR, and GH, with the S clade corresponding to the S type in the Tang study (11–13). Here we present a comprehensive epidemiological and genomic analysis of SARS-CoV-2 genomes from 10 patients in Shaoxing (Zhejiang Province), a mid-sized city about 500 miles away from Wuhan at the early stages of the outbreak.

## Materials and Methods

### Study Design and Ethics

Ten remnant nasopharyngeal swab samples collected between 1/27/2020 and 2/7/2020, and tested positive by a SARS-CoV-2 real-time PCR assay with cycle threshold (C_t_) values of <28, were included in this study. The samples were de-identified except the associated epidemiological data were retained. Since the patient identification was removed and the samples used in this study were remnant and otherwise would be discarded, the Shaoxing Center for Disease Control and Prevention had determined that the institutional review boards (IRB) approval was waived for this project, and the informed consent form was not required.

### SARS-CoV-2 PCR and RNA Sequencing

Total nucleic acid was extracted from the nasopharyngeal swabs using the Total Nucleic Acid Extraction Kit (IngeniGen XMK Biotechnologies, Inc., Zhejiang, China). Real-time PCR was performed by using the IngeniGen XMKbio 2019-nCoV (SARS-CoV-2) RNA Detection kit, which targets the highly specific sequences in the *ORF1ab* and *N* genes of the virus, on the ABI 7500 system (ThermoFisher Scientific, Inc., MA, USA). The RNA libraries were constructed using the Ingenigen XMKbio RNA-seq Library Prep Kit (IngeniGen XMK Biotechnologies, Inc., Zhejiang, China). Briefly, DNase was used to remove residual human DNA and the RNA was fragmented, followed by double-strand cDNA synthesis, end-repair, dA-tailing, and adapter ligation. Sequencing was performed by using the 2 × 75 bp protocol on the Nextseq 550 system (Illumina, Inc., CA, USA). Sequencing data have been deposited to NCBI SRA under BioProject PRJNA638211, and to GISAID with accessions EPI_ISL_463889 and EPI_ISL_463894 to 463902.

### Data Analysis

Quality control and trimming of paired-end reads was performed using custom Python scripts as follows: (1) trim 3′ adapters; (2) trim reads at ambiguous bases; (3) filter reads shorter than 40 bp; (4) filter reads with average quality score <20. Host-derived reads were removed by alignment against the GRCh38.p13 genome reference using bowtie2 (v2.3.4.3) (14) with default parameters. The retained reads were then mapped to 163 published SARS-CoV-2 reference genomes obtained from GISAID (https://www.gisaid.org/CoV2020/, accessed March 2, 2020) by bowtie2 (v2.3.4.3) with default parameters. Snippy (v4.5.0) was used for variant and indel calling, and core SNP alignment against the Wuhan-Hu-1 (NC_045512.2) reference (6), FastTree (v2.1.3) which infers approximately-maximum-likelihood phylogenetic trees (15) was used for tree construction using default parameters, and Figtree (v1.4.4) (16) was used to visualize the resulting phylogenetic tree. HISAT2 (v2.2.0) and StringTie (v2.1.3) were used for RNA-Seq alignment and transcript assembly to identify novel isoforms (17, 18). Additional statistical analyses and visualizations were performed using the “ggplot2” package in the R statistical environment (v3.6). The clade and lineage nomenclature was determined by using pipeline pangolin (https://github.com/hCoV-2019/pangolin) as described previously (13).

## Results

### Epidemiology of Shaoxing Patients

All 10 patients presented with symptoms (fever and cough) consistent with COVID-19 in late January and early February of 2020. The patients can be categorized into two epidemiologic groups with either a travel history to the Hubei province or sick contact with a confirmed case (Table 1). There was one case where we were unable to obtain a travel or exposure history (Shaoxing-8).

**Table 1:** Epidemiological history of the 10 Shaoxing patients.

**ID**	**Age range**	**History of travel or sick contact**	**Date of symptom onset**	**Date of sample collection**
Shaoxing-01	30–39	Family members traveled together to Hubei province (1/15–1/24)	1/24/20	1/27/20
Shaoxing-02	70–79		1/29/20	1/30/20
Shaoxing-03	60–66		1/28/20	1/30/20
Shaoxing-04	50–59		1/29/20	1/31/20
Shaoxing-05	50–59	Traveled to Hubei (1/16–1/23)	1/29/20	1/31/20
Shaoxing-06	30–39	Resident of Wuhan; Traveled to Shaoxing on 1/17	1/29/20	1/31/20
Shaoxing-07	<10	Traveled to Hubei (1/11–1/24); Two family members were confirmed cases	1/30/20	1/30/20
Shaoxing-08	50–59	Unknown	1/31/20	2/7/20
Shaoxing-09 Shaoxing-10	30–39	Family members living together no travel history; contact with a confirmed case from Ningbo, Zhejiang on 1/27 30–39	2/2/20 2/5/20	2/5/20 2/6/20

There are two apparent clusters in these 10 patients. The first cluster involves four patients who are relatives and traveled together to Hubei province for a wedding in late January. The first patient in this cluster had symptom onset on their last day in Hubei province while the other three patients had symptom onset 4–5 days after coming back to Shaoxing (Table 1). The second cluster involves two patients who are family members that live together and did not travel to Hubei province. One of the family members (Shaoxing-09) had a sick contact with a confirmed case who visited her but lived in Ningbo, a more populated city in Zhejiang province (Table 1).

### Metagenomic Sequencing

**Figure 1:**
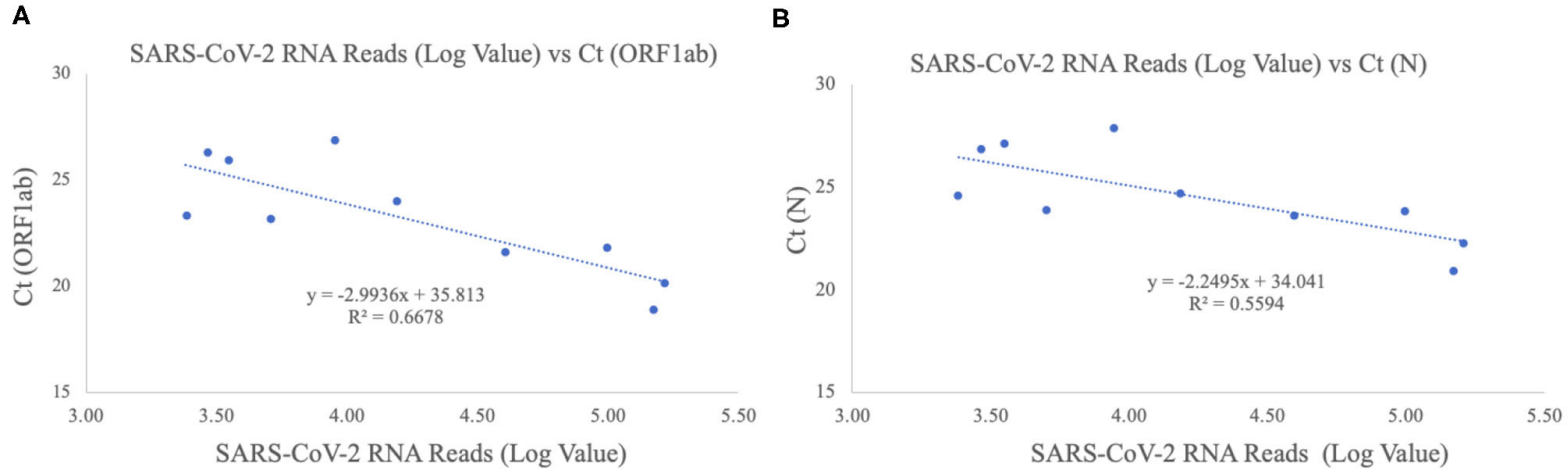
Correlation of C_t_ values and SARS-CoV-2 RNA reads. **(A)** *ORF1ab* gene target. The X-axis plots the log value of the SARS-CoV-2 RNA reads while the Y-axis plots the C_t_ values for the *ORF1ab* gene for the 10 Shaoxing patients. **(B)** *N* gene target. The X-axis plots the log value of the SARS-CoV-2 RNA reads while the Y-axis plots the C_t_ values for the *N* gene for the 10 Shaoxing patients.

The patients were confirmed to have SARS-CoV-2 infection by a commercial Real-time PCR assay. The average C_t_ values for the 10 patient samples were 23.17 for *ORF1ab* and 24.54 for *N* (Table 2). Metagenomic sequencing was performed to recover the full viral genome. The total number of sequence reads per samples ranged from 10.4 to 27.5 million with an average of 17.1 million. A small percentage of these reads mapped to SARS-CoV-2 RNA genome and we did not identify any novel transcripts or fusion events, nor did we detect any insertion or deletion (Table 2). The range of sequence reads that mapped to SARS-CoV-2 RNA was 2,413–163,158 with an average of 49,066. We observed a clear negative correlation between the C_t_ values of each gene (*ORF1ab* and *N*) and the log value of SARS-CoV-2 RNA reads (Figure 1). However, the linearity is not significant (*R*^2^ = 0.6628, 0.5595 for *ORF1ab* and *N*, respectively), indicating that the number of RNA reads measured by metagenomics sequencing are only semi-quantitative and cannot be interpreted directly as viral loads.

**Table 2:** Summary of sequencing results of 10 Shaoxing patient samples.

**ID**	**Ct value (ORF1ab)**	**Ct value (*N*)**	**Total reads (PE 75)**	**2019-nCoV RNA (raw reads)**	**2019-nCoV RNA (log value)**	**Genome coverage (%)**	**Mean depth (X)**
Shaoxing-01	21.57	23.62	17,158,277	40,057	4.60	99.4	219
Shaoxing-02	18.86	20.93	13,602,710	149,682	5.18	99.9	929
Shaoxing-03	20.09	22.25	24,769,343	163,158	5.21	99.8	1,024
Shaoxing-04	24.02	24.68	21,509,477	15,424	4.19	100.0	81
Shaoxing-05	21.81	23.81	14,043,326	99,521	5.00	99.9	591
Shaoxing-06	25.88	27.08	18,909,299	3,535	3.55	99.9	18
Shaoxing-07	23.11	23.87	10,480,051	5,063	3.70	99.8	26
Shaoxing-08	23.34	24.53	11,506,909	2,413	3.38	99.9	12
Shaoxing-09	26.81	27.85	27,517,291	8,897	3.95	99.9	47
Shaoxing-10	26.24	26.8	11,071,595	2,911	3.46	99.7	15
Min	18.86	20.93	10,480,051	2,413	3.38	99.4	12
Max	26.81	27.85	27,517,291	163,158	5.21	100.0	1,024
Mean	23.17	24.54	17,056,828	49,066	4.22	99.8	296

**Figure 2:**
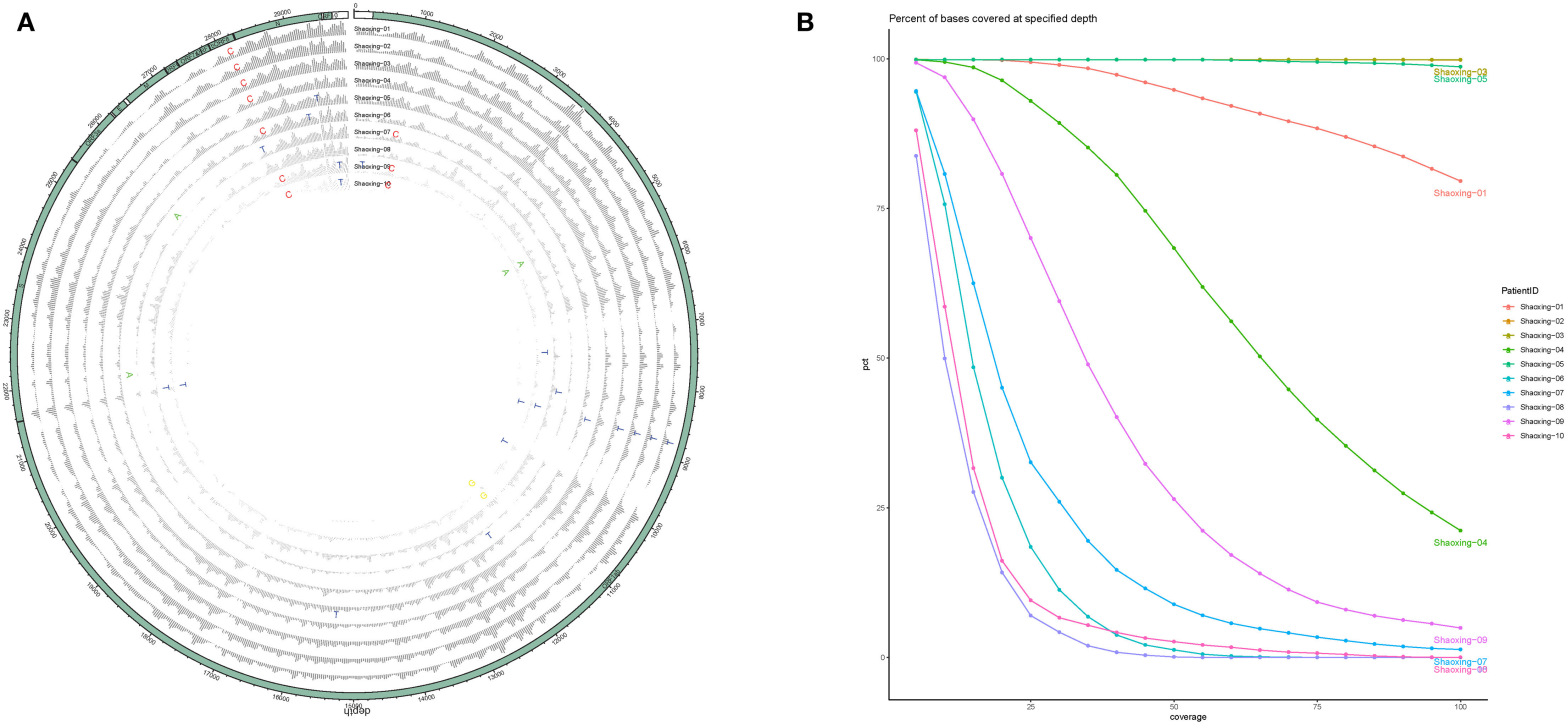
Coverage and depth. **(A)** Coverage and depth map. Normalized depth at each position along the genome are shown for each sample, with SNPs marked by the alternate allele. **(B)** Depth ratio. The X-axis plots the log value of the depth for each genome while the Y-axis plots the cumulative percentage of bases covered to the specified depth.

With a large variation in the SARS-CoV-2 RNA mapped reads, we were still able to obtain excellent coverage and depth when each genome was mapped to the first SARS-CoV-2 genome, Wuhan-Hu-1 [(6); Figure 2A]. The coverage for all genomes was above 99% and the mean depth for the genomes ranged from 12X to 1024X (Table 2, Figure 2B). Genomes sequenced to a relatively low mean depth (12X to 47X) were still able to be genotyped successfully but our results suggest that SARS-CoV-2 read counts of at least 15,000 yield sufficiently high depth to characterize even low prevalence or rare mutations.

### Estimation of Mutation Rate

To determine the single nucleotide polymorphisms (SNPs) of SARS-CoV-2 in these 10 patients, we mapped each genome to the original Wuhan-Hu-1 reference which was collected on December 31, 2019 (6). The genomes contained a fairly moderate number of SNPs (mean of 4 SNPs, range 1-9) (Table 3), consistent with previous reports of relatively low mutation rates (19). The genomes with the largest number of SNPs came from individuals who had contact with a confirmed case from Ningbo, Zhejiang and no travel history to the Hubei province (Table 3, Shaoxing-9 and 10).

**Table 3:** Summary of genomic descriptions for the Shaoxing SARS-CoV-2 genomes.

**ID**	**Clade**	**No. of SNP^a^**	**No. of Days^b^**	**Mutation rate (#SNP/day)**	**Mutation rate (#SNP/day/nt)**	**Mutation rate (#SNP/yr/nt)**
Shaoxing-01	S	2	27	0.07	2.48E-06	9.04E-04
Shaoxing-02	S	2	30	0.07	2.23E-06	8.14E-04
Shaoxing-03	S	2	30	0.07	2.23E-06	8.14E-04
Shaoxing-04	S	2	31	0.06	2.16E-06	7.87E-04
Shaoxing-05	L	2	31	0.06	2.16E-06	7.87E-04
Shaoxing-06	S	3	31	0.1	3.24E-06	1.18E-03
Shaoxing-07	L	5	30	0.17	5.57E-06	2.03E-03
Shaoxing-08	L	1	38	0.03	8.80E-07	3.21E-04
Shaoxing-09	S	9	36	0.25	8.36E-06	3.05E-03
Shaoxing-10	S	9	37	0.24	8.13E-06	2.97E-03
Min		1	27	0.03	8.80E-07	3.21E-04
Max		9	38	0.25	8.36E-06	3.05E-03
Mean		4	32	0.11	3.74E-06	1.37E-03

a *SNP calculated by mapping each genome to the genome of Wuhan-Hu-1 (NC_045512.2) (6)*.

b *Number of days between the date that the sample was collected and the date the Wuhan-Hu-1 sample was collected (12/31/2019)*.

Using the SNP analysis, we calculated the various mutation rates using the number of days between the date that the sample was collected and the date the Wuhan-Hu-1 sample was collected. The mutation rate (SNP per day) ranged from 0.03 to 0.25 (Table 3). We used this mutation rate to calculate the nucleotide substitution per site per day and the nucleotide substitution per site per year. We saw an average mutation rate of 3.74 × 10^−6^ nucleotide substitution per site per day and an average mutation rate of 1.37 × 10^−3^ nucleotide substitution per site per year (Table 3).

We investigated each SNP to determine if there were any non-synonymous mutations in genes important to the virus lifecycle. Several non-synonymous mutations were found in the following genes: *orf1ab, orf3, N, orf8*, and *orf10* (Table 4A). These mutations were identified with sufficient confidence as at least 5X depth was achieved in all the SNPs (Table 4B, highlighted in orange), and at least 4X depth was achieved at the positions of these SNPs in all samples (Table 4B, unhighlighted). In 7 samples, we identified C8782T and T28144C mutations which are the landmark events of the S clade (Table 4A). We did not identify other GISAID clade defining mutations outlined by Mercatelli and Giorgi (12). No non-synonymous mutations were found in the S gene, which encodes the spike protein that's critical for viral binding to human receptor ACE2 (9). Notably in the cluster of the two family members (Shaoxing-9 and−10), the two viruses are closely related but not identical. Shaoxing-9 was infected first and then transmitted to Shaoxing-10, whose virus gained a non-synonymous mutation C9962T in the ORF1ab gene (Table 4). This could be explained by the sequential transmission, however, we could not rule out a possibility of intra-host viral heterogeneity in the two patients.

**Table 4A:** Summary of SNPs in the 10 SARS-CoV-2 genomes.

**SNP#**	**Position**	**Gene**	**Reference nt**	**MutationString**	**MutationString2**	**Shaoxing-01**	**Shaoxing-02**	**Shaoxing-03**	**Shaoxing-04**	**Shaoxing-05**	**Shaoxing-06**	**Shaoxing-07**	**Shaoxing-08**	**Shaoxing-09**	**Shaoxing-10**
1	207	Non-coding	C	NA	NA									T (non-coding)	
2	889	orf1ab	T	A208A	orf1ab:A208A							C (A)			
3	946	orf1ab	T	G227G	orf1ab:G227G									C (G)	C (G)
4	5099	orf1ab	T	S1612T	orf1ab:S1612T									**A (S->T)**	**A (S->T)**
5	7420	orf1ab	C	I2385I	orf1ab:I2385I									T (I)	T (I)
6	8344	orf1ab	C	D2693D	orf1ab:D2693D								T (D)		
7	8782^*^	orf1ab	C	S2839S	orf1ab:S2839S	T (S)^*^	T (S)^*^	T (S)^*^	T (S)^*^		T (S)^*^			T (S)^*^	T (S)^*^
8	9962	orf1ab	C	H3233Y	orf1ab:H3233Y										**T (H->Y)**
9	11430	orf1ab	A	Y3722C	orf1ab:Y3722C									**G (Y->C)**	**G (Y->C)**
10	11916	orf1ab	C	S3884L	orf1ab:S3884L							**T (S->L)**			
11	15324	orf1ab	C	N5020N	orf1ab:N5020N					T (N)					
12	21676	S	C	Y38Y	S:Y38Y									T (Y)	T (Y)
13	22081	S	G	Q173Q	S:Q173Q							A (Q)			
14	25672	ORF3a	C	L94I	ORF3a:L94I							**A (L->I)**			
15	28000	ORF8	C	P36L	ORF8:P36L							**T (P->L)**			
16	28144^*^	ORF8	T	L84S	ORF8:L84S	C (L->S)^*^	C (L->S)^*^	C (L->S)^*^	C (L->S)^*^		C (L->S)^*^			C (L->S)^*^	C (L->S)^*^
17	29095	N	C	F274F	N:F274F						T (F)				
18	29303	N	C	P344S	N:P344S					**T (P->S)**					
19	29625	ORF10	C	S23F	ORF10:S23F									**T (S->F)**	**T (S->F)**
									Non-Synonymous	Synonymous					

*SNP analysis was based on NC_045512.2 (Wuhan-Hu-1) as the reference genome (6). Variants are denoted as nucleotides vs. the reference base. Amino acid changes listed in parentheses with synonymous mutations listed as a single residue. The non-synonymous mutations are bolded*.

* *Mutations of the S clade*.

**Table 4B:** Depth of SNP in the 10 SARS-CoV-2 genomes.

**Position**	**MutationString**	**Shaoxing-01**	**Shaoxing-02**	**Shaoxing-03**	**Shaoxing-04**	**Shaoxing-05**	**Shaoxing-06**	**Shaoxing-07**	**Shaoxing-08**	**Shaoxing-09**	**Shaoxing-10**
207	NA	147	421	643	65	380	11	20	5	17	7
889	A208A	363	1,091	1,666	177	1,018	21	44	19	74	21
946	G227G	166	453	746	80	434	12	24	5	45	17
5099	S1612T	192	617	784	60	575	8	23	7	49	10
7420	I2385I	97	384	403	34	211	5	9	6	10	5
8344	D2693D	161	492	598	63	350	20	4	12	22	11
8782	S2839S	319	948	1,102	108	697	18	29	9	43	20
9962	H3233Y	285	973	1,085	116	796	23	33	23	60	13
11430	Y3722C	180	642	642	78	336	12	18	16	39	17
11916	S3884L	170	698	761	80	444	15	12	21	28	13
15324	N5020N	232	840	877	92	528	22	14	15	35	14
21676	Y38Y	246	1,010	941	105	543	30	36	12	62	14
22081	Q173Q	157	841	694	56	347	15	7	17	41	8
25672	L94I	124	557	476	41	328	7	15	7	37	11
28000	P36L	313	1,112	1,090	113	814	33	57	25	106	44
28144	L84S	189	815	728	79	495	29	44	21	53	19
29095	F274F	386	1,271	1,331	106	798	29	81	25	105	34
29303	P344S	497	1,438	1,452	168	1,067	43	128	36	173	67
29625	S23F	296	1,053	1,004	108	618	34	62	26	100	30

*Orange highlighted cells indicate detected mutations and the unhighlighted cells indicate wild-type*.

### SARS-CoV-2 Genotype and Phylogenetic Characteristics

**Figure 3:**
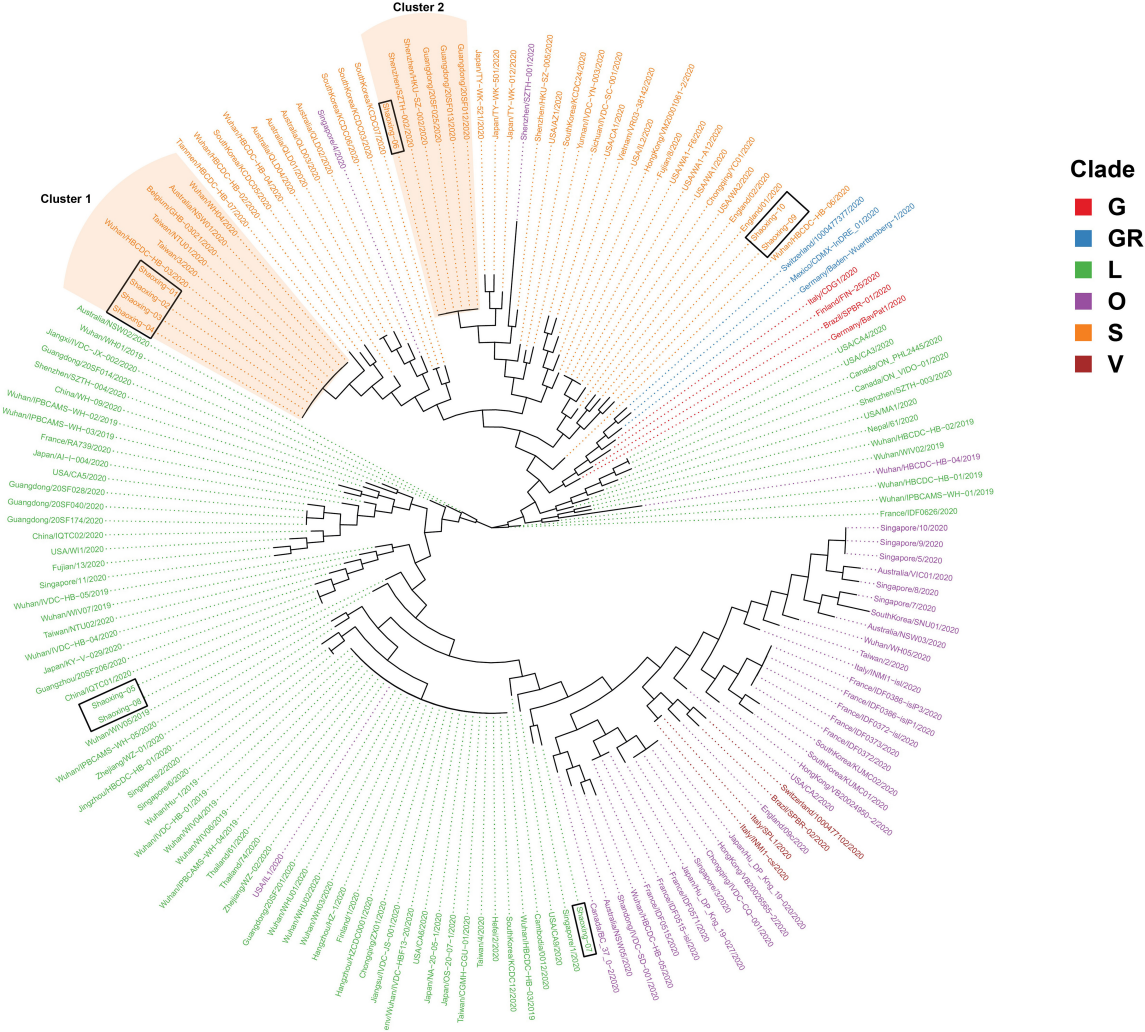
Phylogenetic comparison of SARS-CoV-2 genomes. Phylogenetic comparison of 163 published genomes from GISAID (8) and the 10 Shaoxing genomes (boxed out). Genomes are color-coded based on their clade.

Previous reports demonstrate that SARS-CoV-2 has two genotypes known as L type and S type in the early phase of the outbreak (10); however, recent classification has divided the SARS-CoV-2 genomes into 7 different clades (O, S, L, V, G, GR, GH) (12). We decided to compare our 10 SARS-CoV-2 genomes to 163 other SARS-CoV-2 genomes obtained from GISAID (8) published by mid-March. Although the majority of the SARS-CoV-2 genomes obtained from GISAID in the early phase of the pandemic belonged to the L clade (Figure 3, green), the 10 Shaoxing SARS-CoV-2 genomes (Figure 3, black boxes) were distributed throughout these genomes with more of them classified in the S clade (Figure 3, orange). The more detailed phylogenetic tree with bootstrap values is shown in Supplementary Figure 1.

Interestingly, four of the Shaoxing SARS-CoV-2 genomes (Shaoxing−1 to−4) were identical to six other GISAID SARS-CoV-2 genomes (Figure 3, Cluster 1). These six other genomes were isolated from patients all over the world: two from Wuhan, two from Taiwan, one from Belgium, and one from Australia (Figure 3, Cluster 1). Shaoxing-6 is identical to five other genomes isolated in Shenzhen, Guangdong Province in Southern China (Figure 3, Cluster 2). Notably, in all 10 Shaoxing patients, we found no virus with D614G Spike gene mutation, which was shown to start spreading in Europe in early February, and rapidly become the dominant form in the rest of the world out of China (20).

## Discussion

In this study, we sequenced the SARS-CoV-2 genome from 10 patient samples from Shaoxing, Zhejiang, China. Using metagenomic sequencing, we were able to obtain above 99% coverage and an average depth of 296X for all 10 SARS-CoV-2 genomes. Although not statistically significant, there does appear to be a clear negative correlation between the C_t_ values of both gene targets and the log count of SARS-CoV-2 RNA sequence reads acquired by metagenomics sequencing. This suggests that the log value of RNA sequence reads by metagenomics sequencing may be used as a semi-quantitative measurement for SARS-CoV-2 viral loads.

The rapid spread of this virus is highlighted by the fact that four SARS-CoV-2 genomes from Shaoxing individuals were identical to six other SARS-CoV-2 genomes from patients all over the world. Our data support recent publications that the virus had spread rapidly around the world especially in Europe before the United States (21–23).

Overall, we did not see a large number of SNPs in these SARS-CoV-2 genomes. The greatest number of SNPs seen was 9 and these two SARS-CoV-2 genomes were from individuals with no travel history to Hubei province (Table 3, Shaoxing-9 and 10). Instead, Shaoxing-9 and 10 had contact with a confirmed case from Ningbo, another city outside of Hubei. We can use these data to infer that the virus accumulated more mutations when it was spread to another city outside of Hubei first before coming to Shaoxing, compared to the virus from people traveled to Shaoxing directly from Hubei.

We combined epidemiologic data with the SNP analysis to estimate the mutation rate of the SARS-CoV-2 from these 10 patients. We saw an average mutation rate of 1.37 × 10^−3^ nucleotide substitution per site per year for SARS-CoV-2, which is consistent with other reports on the mutation rate of SARS-CoV-2(19, 24) and SARS-CoV-1 with a reported mutation rate of 0.80–2.38 × 10^−3^ nucleotide substitution per site per year (25). These data demonstrate that SARS-CoV-2 is similar in the mutation rate as other coronaviruses.

The major limitation of this study is that we only had 10 samples analyzed due to the requirement of sufficient SARS-CoV-2 RNA from a metagenomic sample. However, with the development of SARS-CoV-2 probe enrichment or multiplex PCR protocols, this type of viral sequencing analysis may be applied to samples with lower viral loads, thereby enabling more complete molecular epidemiological surveillance. In addition, the C_t_ value cut-off of 28 established in this study may not be directly applicable to other real-time PCR assays due to the technical differences. Inevitably, exclusion of samples with lower viral load could introduce bias in the genomic surveillance of SARS-CoV-2 and potentially lead to missed identification of important genotypes. Last, we did not analyze or predict the potential biological changes that may be caused by the identified mutations, such as alterations in RNA secondary structure, protein stability, interaction with host proteins, and codon usage, etc.

In summary, we demonstrated that a full viral genomic analysis is feasible via metagenomics sequencing directly on nasopharyngeal samples, which allows retrospective molecular surveillance on SARS-CoV-2 to understand the dynamics of the outbreak in the early phase. The identical virus found in patients in Shaoxing, a mid-sized city outside of Hubei, China, and patients in Europe and Australia was striking. Our analysis added to the growing body of evidence that SARS-CoV-2 spread extremely quickly around the globe as early as January. Although only 10 patients were included in this study, we found numerous mutations (both synonymous and non-synonymous) across the entire viral genome. Our study contributed to the understanding of the SARS-CoV-2 evolution in the early phase of the COVID-19 pandemic.

## Data Availability Statement

The datasets presented in this study can be found in online repositories. The raw FASTQ files have been deposited to NCBI SRA under BioProject PRJNA638211, and the snippy-based consensus genome sequences have been deposited to GISAID. They can be found with accessions EPI_ISL_463889 and EPI_ISL_463894 to EPI_ISL_463902.

## Ethics Statement

Ethical review and approval was not required for the study on human participants in accordance with the local legislation and institutional requirements. Written informed consent from the participants' legal guardian/next of kin was not required to participate in this study in accordance with the national legislation and the institutional requirements.

## Author Contributions

JC, EH, FL, ZL, JT, and SY conceived and planned the experiments. JC, ZJ, QZ, JW, and YW carried out the experiments. EH, HW, and FL performed the data analysis. JC and EH took the lead in writing the manuscript. ZL, JT, and SY supervised the research project. All authors provided critical feedback and helped shape the research, analysis and manuscript.

## Conflict of Interest

HW and YW were employed by the company Shaoxing IngeniGen XMK Biotechnologies. FL was the Chief Executive Officer of the company Three Coin Analytics. The authors declare that this study received funding from Shaoxing IngeniGen XMK Biotechnologies. The funder had the following involvement with this study: providing sequencing data and preliminary bioinformatics analysis. The remaining authors declare that the research was conducted in the absence of any commercial or financial relationships that could be construed as a potential conflict of interest.

## Supplementary Material

Supplementary Figure 1 Phylogenetic tree of SARS-CoV-2 genomes with bootstrap values.
