# Supplementary figures and images for: Epidemiological and Genomic Analysis of SARS-CoV-2 in 10 Patients From a Mid-Sized City Outside of Hubei, China in the Early Phase of the COVID-19 Outbreak
Source: Front Public Health. 2020 Sep 18;8:567621. doi: 10.3389/fpubh.2020.567621 (PMC7531217; doi:10.3389/fpubh.2020.567621)

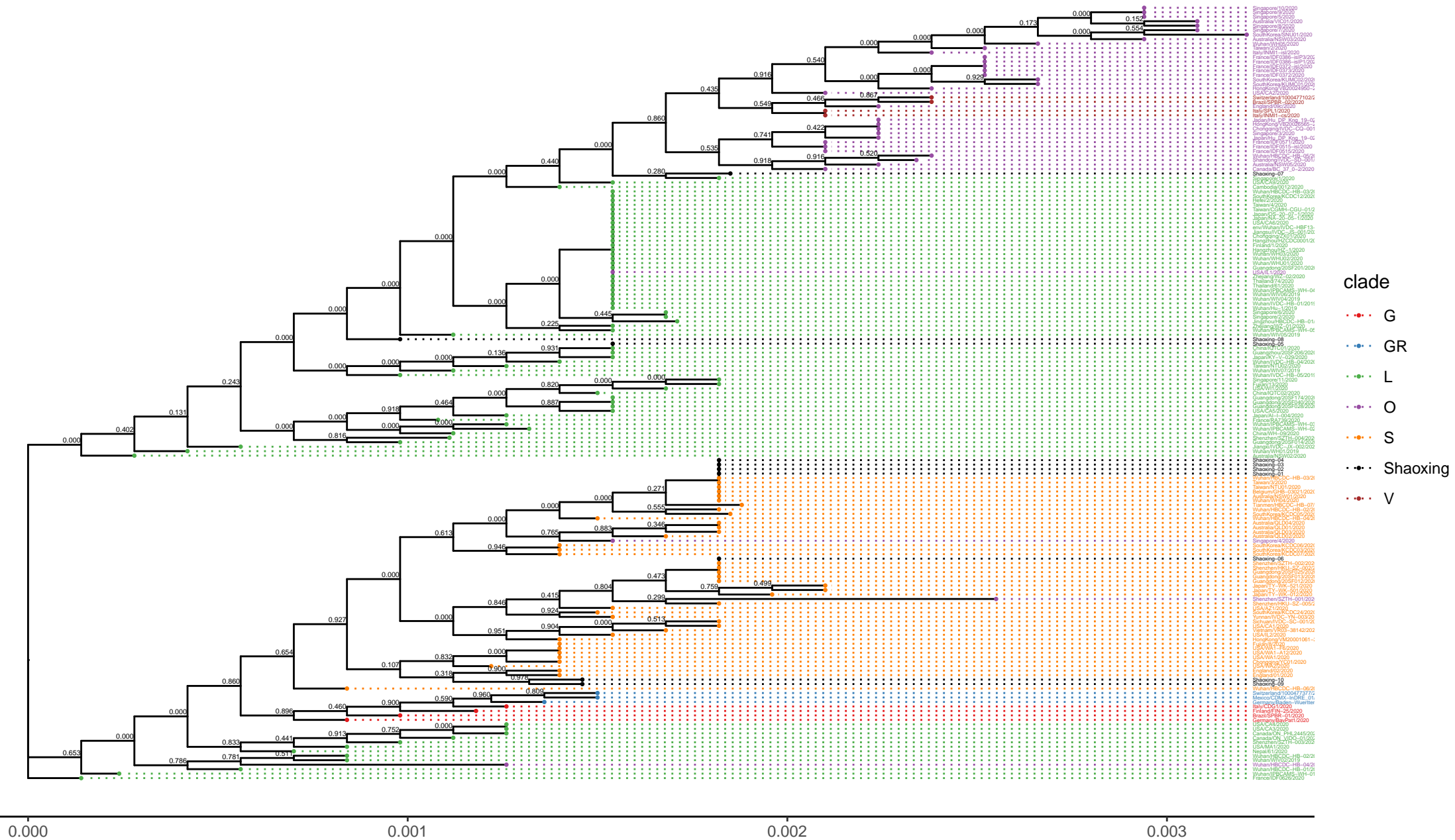

Supplement: Supplementary Figure 1 — Phylogenetic tree of SARS-CoV-2 genomes with bootstrap values. [file Data_Sheet_1.PDF]
